# Calcium signaling is required for anterior patterning in the mouse embryo

**DOI:** 10.1371/journal.pbio.3003430

**Published:** 2025-10-06

**Authors:** Matthew J. Stower, Richard C. V. Tyser, Shifaan Thowfeequ, Felix Zhou, Marta Portela, Konstantinos Miti, Jacintha Sugnaseelan, Xin Lu, Shankar Srinivas

**Affiliations:** 1 DPAG, Institute of Developmental and Regenerative Medicine, University of Oxford, Oxford, United Kingdom; 2 Lyda Hill Department of Bioinformatics, University of Texas Southwestern Medical Center, Dallas, Texas, United States of America; 3 Cecil H. & Ida Green Center for System Biology, University of Texas Southwestern Medical Center, Dallas, Texas, United States of America; 4 Ludwig Institute for Cancer Research, University of Oxford, Oxford, United Kingdom; The University of Edinburgh School of Biological Sciences, UNITED KINGDOM OF GREAT BRITAIN AND NORTHERN IRELAND

## Abstract

Anterior–posterior axis formation in the mouse embryo requires the active migration of the DVE cell population at E5.5. While intracellular Ca^2+^ signaling has been shown to control cell migration in multiple cell contexts, it is unknown whether it is required for DVE migration. The pattern of Ca^2+^ activity in the mouse embryo at early peri-implantation stages is also unknown. Using the GCaMP6f Ca^2+^ reporter line, we performed a detailed assessment of Ca^2+^ dynamics between E0.5 and E5.5 using live imaging. We find that prior to implantation, Ca^2+^ transients are rare, but at E5.5 widespread, periodic, Ca^2+^ transients in extraembryonic tissues can be observed, including in the VE and ExE. In contrast, cells of the E5.5 epiblast remain relatively quiescent but show sporadic large-scale multicellular waves. Inhibition of SERCA at E5.5 abolishes Ca^2+^ transients and leads to DVE arrest, indicative that these transients are required for axial patterning. Together, these results reveal the pattern of Ca^2+^ handling in the early mouse embryo and a novel requirement in anterior–posterior axis formation.

## Abstract 2

During embryonic development, distal visceral endoderm (DVE) cells undergo migration to shape anterior-posterior axis specification. This study reveals the role of calcium signaling in this context, providing a detailed assessment of calcium dynamics and showing that inhibition of calcium transients cause DVE arrest.

## Introduction

The distal visceral endoderm (DVE) is a specialized cell population in the embryonic day 5.5 (E5.5) mouse embryo that undergoes a collective migration required for anterior–posterior axis specification [1–3]. DVE cells, induced at the distal tip of the E5.5 egg-cylinder, migrate over the course of 3–5 h [2] toward one side of the embryo where they secrete inhibitors of the TGF-β/NODAL [4] and WNT [5] pathways that are required for primitive streak formation. The primitive streak is the site of gastrulation and beings to form from ~E6.5 on the side of the epiblast farthest from the inhibitory signals secreted by the DVE [6,7]. Mutants where the DVE is not induced, or in which DVE migration is aberrant or inhibited lead to incorrectly patterned, nonviable embryos [1,6,8,9].

DVE cells are a migratory epithelial cell population that retain intact tight- and adherent-junctions, defined by ZO-1 and E-cadherin [10], throughout their migration. However, they also show characteristics traditionally associated with mesenchymal cells—highly dynamic basally located projections polarized in the direction of migration [2,11]. As DVE cells remain within the monolayer epithelium of the visceral endoderm (VE), they must actively regulate both apical and basal domains throughout migration.

Regulation of intracellular calcium (Ca^2+^) signaling is involved in a diverse array of cellular responses, including fertilization cell migration, proliferation, apoptosis, and differentiation [12–15]. Cells can generate a transient cytosolic increase in Ca^2+^ through mobilization from intracellular stores (including the endoplasmic reticulum (ER), Golgi apparatus and mitochondria), or from extracellular sources [12]. This is controlled by ion pumps and channels, including the sarcoplasmic/endoplasmic reticulum Ca^2+^ ATPase (SERCA) [16]. Cells can also signal cell-to-cell via gap junctions [17], causing a sequential wave of Ca^2+^ transients that can span multiple cells in a tissue [18]. Intracellular Ca^2+^ can regulate migration by influencing the cytoskeleton, cell polarity, and focal adhesions [19]. For example, in mesenchymal cells, Ca^2+^ can form a front-rear (high-low) gradient, polarizing cells to form lamellipodia only at the front of the cell while mediating rear detachment of protrusions through regulation of focal adhesions [19,20]. In some cases, such as in *Xenopus* axial mesoderm, no intracellular gradient is observable, but Ca^2+^ is elevated in the leading cells of the migratory cell population [21].

In the mouse oocyte, sperm entry triggers a transient cytosolic release of Ca^2+^ within 3 min, followed by a series of Ca^2+^ oscillations every 20–30 min over the course of several hours [14,22–24]. This is an event highly conserved across vertebrate taxa, though species differ in the number and frequency of subsequent oscillations [25–27]. In the mouse 2-cell stage, it has been shown that small vesicles located at the sub-plasmalemmel cytoplasm, termed Membrane-Associated RNA-containing Vesicles (MARVs) have a higher level of Ca^2+^ than the cytosol [28] and persist until embryonic day 3.5 (E3.5) [28]. MARVs act as a critical Ca^2+^ reservoir for the regulation of mitochondrial activity, and transfer Ca^2+^ from membrane-located MARVs to the mitochondria that surround the nucleus at these early stages, though no cytoplasm-wide Ca^2+^ transients have been reported [28].

After this stage, our knowledge of Ca^2+^ signaling in early vertebrate development comes mainly from studies in fish, amphibians, and chick [21,29–34]. Ca^2+^ oscillations have only been observed in the mouse node at E7.5 [35] and the forming cardiac crescent from E7.75 [36], constituting evidence for continued Ca^2+^ transients during early mouse development [14]. Therefore, there exists a gap in our knowledge of any role of Ca^2+^ in the mouse at early stages.

It has been possible to visualize Ca^2+^ transients during early development using fluorescent Ca^2+^ indicators such as f-aequorin [37], Calcium Green-1 dextran [29], and Fluo-4 AM [28]. However, there are technical difficulties to these approaches in the mouse embryo, particularly as it grows, including the dilution of injected reporters as cells divide, and the necessity for diffusion of the dye throughout the depth of tissues. To circumvent these issues, we made use of a genetically encoded fluorescent Ca^2+^ reporter mouse line GCaMP6f [38,39] to perform a detailed stage-series assessment of Ca^2+^ dynamics between E0.5 and E5.5 using timelapse microscopy. We find that at up to E3.5 Ca^2+^ oscillations are rare, stochastic events, while at E4.5 there is a higher level of Ca^2+^ in the trophectoderm and primitive endoderm compared to the epiblast. At E5.5, there is a significant up-regulation in the frequency and amplitude of oscillations, mainly in extraembryonic tissues (ExE and VE). The epiblast remains relatively quiescent, but we observe occasional multicellular Ca^2+^ waves that spread across this tissue. Inhibition of SERCA abolishes Ca^2+^ transients at E5.5 and causes the arrest of DVE migration, an event required for anterior–posterior axis formation.

## Methods

### Ethics statement

All experiments were approved by Oxford University Departmental Animal Welfare and Ethical Review Board (AWERB) and were performed under UK Home Office Licence Project 30/3420 Protocol 03 Breeding and Maintenance of genetically altered animals (Mild), and UK Home Office Licence Project PP7051082 Protocol 05 Breeding and Maintenance of GA animals (Mild).

### Mouse strains

B6;129S-*Gt(ROSA)26Sor*^*tm95.1(CAG-GCaMP6f)Hze*^/J [39] that contain a Cre-inducible GCaMp6f reporter were crossed to B6.Cg-*Edil3*^*Tg(Sox2-cre)1Amc/*^J, Sox2-Cre [40] mice to generate mice that constitutively express the GCaMP6f reporter. Offspring were bred to subsequently segregate out the Sox2-Cre transgene and establish a constitutive, ubiquitous GCaMP6f reporter line. GCaMP6f homozygous stud males were crossed with *Gt(ROSA)26Sor*^*tm4(ACTB-tdTomato,-GFP)Luo*^ mT/mG [41] or CD1 (Charles River) females to generate embryos for experiments. mT/mG mice ubiquitously express tdTomato fused to a mutated 8 amino-acid region of the MARKS protein that act as a myristolation and palmitoylation site that target it to the cell membrane [42]. For long-term culture experiments, we used the Hex-GFP mouse line [43] where DVE cells are marked by cytoplasmic EGFP. Homozygous Hex-GFP stud males (with and without the mT/mG allele) were mated with CD1 females (Charles River). All mice were maintained on a 12 h light, 12 h dark cycle. Noon on the day of finding a vaginal plug (0.5 days *post coitum*) was designated embryonic day (E)0.5. Embryos of the appropriate stage were dissected in M2 medium (Sigma) with fine forceps and tungsten needles as previously described [44].

### Confocal microscopy—Live imaging

Dissected embryos were transferred to an 8-well Lab-Tek II chambered cover-glass slide (Sigma) with 250 μl of culture medium (1:1 knock-out serum replacement: CMRL, supplemented with 1% L-glutamine) pre-equilibrated at 37 °C and 5% CO_2_. The imaging slide was mounted into a pre-warmed stage and chamber of an inverted, ZIESS LSM 880 confocal microscope. To establish imaging parameters, we tested a range of settings (including varying laser power, exposure, summing, and increasing the width of the confocal pin-hole), to confirm that we were not missing any weak expression of the GCaMP transgene. A list of the final microscope settings, imaging duration, and intervals are included herein (see S1 Table). For more detailed analysis of Ca^2+^ dynamics in E5.5 embryos, a single mid-sagittal z-section was imaged at an interval of 5 s for a duration of 10 min using a water immersion 40×/1.2 NA objective. Each developmental stage comprised a freshly dissected litter.

### Light-sheet microscopy—Zeiss Z.1 microscope

For light-sheet imaging experiments, 20 μl glass capillaries (Brand, 701904) with plungers were used to create a 2% agarose cylinder, in which a copper wire with a diameter of 150 μm was inserted. Once the agarose solidified, the wire was removed to leave a hollow lumen into which an embryo could be carefully inserted. The glass capillary was then mounted into the imaging chamber of a ZIESS Z1 light-sheet microscope, and the region of the agarose cylinder containing the mounted embryo extruded into an imaging chamber filled with pre-heated culture medium (as above). Imaging was performed using a 63×/1.0 NA Plan-Apochromat water immersion objective with dual-side illumination. Z-stack volumes were then captured at a 10-s interval for 10 min (see S1 Table). Extended focus projections were generated by ZEN software (ZEISS).

### Lattice light-sheet microscopy (LLSM)

For LLSM, a ZEISS Lattice Light-sheet 7 microscope equipped with a 13.3 × 0.4 NA illumination objective, 44.83 × 1.0 NA water immersion detection objective with an Alvarez manipulator and a pre-defined Sinc3,100 µm × 1,800 nm light sheet beam was used. The heating and CO_2_ concentrations were maintained at 37 °C and 5%, respectively, using the IncuControl system (Version 1.0.3; Ibidi). Embryos were stabilized between two glass rods within the chamber of an 8-chambered #1.5 µ-slide (Ibidi) filled with culture medium (as above), so that the light-sheet scans through the embryo along its anterior–posterior axis. X-stack volumes were obtained at 0.8 µm x-intervals at imaging intervals of 2 s for 20 min (to visualize intracellular Ca^2+^ signal propagation), 20 s for 5 h (to image Ca^2+^ waves), or 5 min for 6 h (for DVE migration live imaging). The resulting image stacks were deskewed with coverglass correction using the ZEN Blue Pro software (Version 3.10, ZEISS).

### Pharmacological inhibitor experiments

GCaMP6f embryos from one or more litter were randomly separated into wells of an 8-well Lab-Tek II chambered cover-glass slide filled with culture medium (as above). For short-term inhibitor experiments, each embryo was imaged at a single mid-sagittal z-level for 2 min duration, with a 10-s interval between images on a pre-heated, climate-controlled chamber (37°C 5% CO_2_) of a ZIESS LSM 880 confocal microscope. An initial timelapse represented a baseline number of Ca^2+^ transients for each embryo (the sum of all transients in all tissues in the timelapse). Next, the selected concentration of inhibitor (10 nM thapsigargin or 200 nM cyclopiazonic acid [CPA]) or control solvent (Dimethyl sulfoxide [DMSO]) was added to each well, and embryos were cultured for 30 mins. Embryos were then re-imaged and the number of Ca^2+^ transients per embryo calculated. Data for each inhibitor comprised a minimum of 3 experimental repeats with multiple embryos in each experiment. For CPA experiments, an additional recovery time point was acquired after washing off the inhibitor and culturing for an additional 30 min in fresh culture medium. Embryos were re-imaged and the total number of Ca^2+^ transients calculated. For long-term culture experiments, homozygous Hex-GFP stud males were crossed with CD1 females, and embryos dissected at E5.5. Embryos from one or more litter were randomly separated into wells of an 8-well Lab-Tek II chambered cover-glass slide filled with culture medium (as above) and imaged on an LSM 880 (ZEISS) confocal microscope. Images of each well were taken before 10 nM thapsigargin or DMSO was added. Embryos were then cultured for 8 hours, then re-imaged, and the number of embryos where the DVE had migrated was scored. Quantifications comprised data from a total of 5 independent experiments.

### Automated analysis of Ca^2+^ transients in E5.5 embryos

Supervised machine learning using a convolutional neural network (CNN) classifier was used to detect the peaks of cytosolic Ca^2+^ transients in the GCaMP6f timelapse data of E5.5 embryos. To train the CNN, a training dataset was created by manually identifying the time point of the peak of Ca^2+^ transients in three E5.5 timelapse datasets. These time points were used to construct an idealized probability Ca^2+^ imaging trace where the intensity is nonzero only at the peak time point. Given an input Ca^2+^ imaging trace, and its idealized equivalent a CNN was trained to predict the probability of observing a Ca^2+^ transient peak within a symmetrical temporal window of *N* = 35 time points. All raw GCaMP6f timelapse data (Ca^2+^ trace) were first de-trended using asymmetric least squares fitting [45] prior to CNN input. Unbiased clustering of Ca^2+^ transients per embryo was carried out to assess the similarity of Ca^2+^ dynamics in each embryo in an unbiased manner. Individual Ca^2+^ traces were pairwise compared to construct a similarity matrix with similarity was defined as the largest positive value of the signal cross-correlation. A hierarchical density-based clustering method (HDBSCAN), was then applied using the Python HDBSCAN package [46] to the similarity matrix to cluster Ca^2+^ traces. Global clustering across all embryos was performed by computing the similarity matrix pooling together all Ca^2+^ traces from all embryos. Individual traces have different minimum and maximum intensities within individual embryos. Traces were first normalized per embryo by z-score, using the mean and standard deviation of all traces from the respective embryo. HDBSCAN generates the optimal clustering number given min_cluster_size – the minimum number of samples in a group for that group to be considered a cluster and min_samples—the number of samples in a neighborhood for a sample to be considered as a core point. The output clusters include ‘core’ clusters, whereby traces within a ‘core’ cluster have high intra-similarity and a ‘outlier’ cluster, which contain traces determined insufficiently similar to a trace with core clusters, and insufficient intra-similarity to form a new core cluster. This approach compares similarity based on the shape of Ca^2+^ traces and is therefore tolerant to slight misalignment in peak times, and peak-to-peak distances. For the global clustering, qualitative examination of the outlier cluster showed traces had a similar spiking pattern but were asynchronous. Therefore, we also used the ‘outlier’ class as a cluster in the global clustering result. We label the clusters numerically, in ascending order of the mean transient number of traces in the cluster. Therefore, in our global clustering, cluster 2 is also the outlier class (Fig 3C). As quantitative measures of Ca^2+^ peak behavior, we computed the mean number of peaks, mean intensity of peaks, periodicity as the mean peak-to-peak distance, and the mean duration of peaks within a trace. Peak duration was measured per peak as the difference between start and end peak times. We define peak start time as the time point just before half peak intensity and end time as the time point just after half peak intensity.

**Fig 1:**
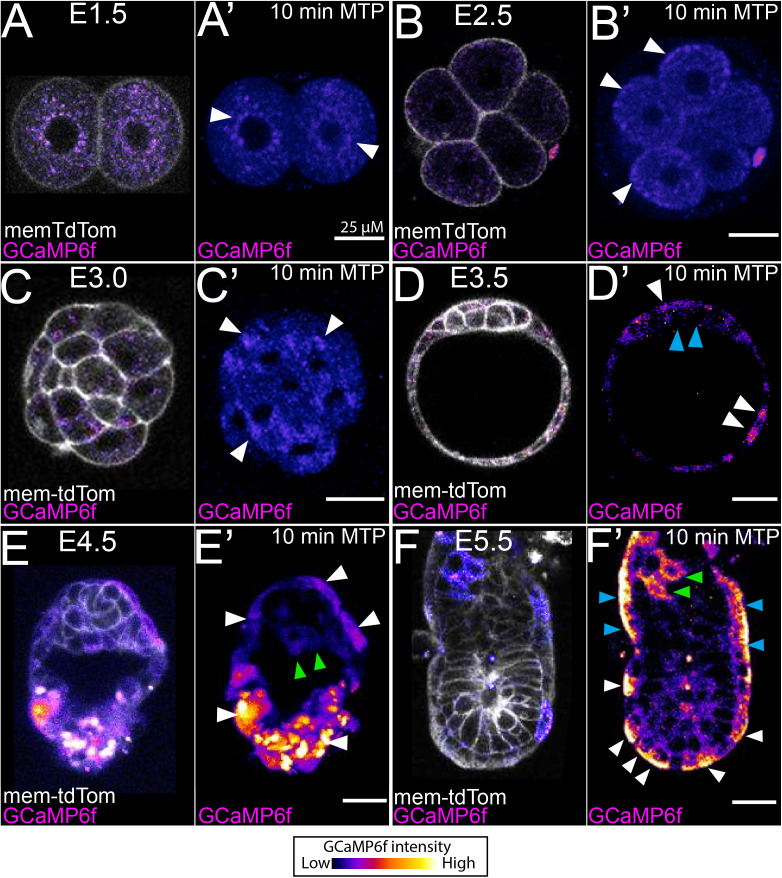
Cytosolic Ca^2^^+^ oscillations occur at post-implantation stages in the mouse embryo. Representative single z-sections of a single-time point, and 10-min max-intensity time projections (MTP) from timelapse imaging of the Ca^2+^ reporter mouse GCaMP6f crossed with membrane-tdTomato at; **(A–A′)** E1.5 **(B–B′)**, E2.5 **(C–C′)**, E3.0 **(D–D′)**, E3.5 **(E–E′)**, E4.75 and (F-F’). **(A–A′)** E5.5. At E1.5, embryos show puncta with a high GCaMP6 signal around the nucleus (A**′**, arrowhead). **(B–B′)** E2.5 show puncta with a high level of Ca^2+^ at the apical domain relative to the rest of the cell (arrowheads in B’). **(C–C′)** E2.5 embryos show large groups of puncta with a high GCaMP6 signal but no cytosolic transients (arrowheads in C’). **(D–D′)** At E3.5, trophectoderm cells show a higher level of Ca^2+^ (white arrowheads) than the ICM (blue arrowheads). **(E–E′)** E4.5 shows higher level of Ca^2+^ in the trophectoderm (white arrowheads in E′) and primitive endoderm (green arrowheads in E′) than the epiblast. **(F–F′)** At E5.5, widespread Ca^2+^ oscillations with high intensity occur in the emVE (white arrowheads in F′) exVE (blue arrowheads in F′), and ExE (green arrowheads in F′). Each stage represented a fresh dissection. Additional timelapse imaging durations and intervals were also tested (see S1 Table and S1–S9 Movies). All scale bars = 25 μM.

**Fig 2:**
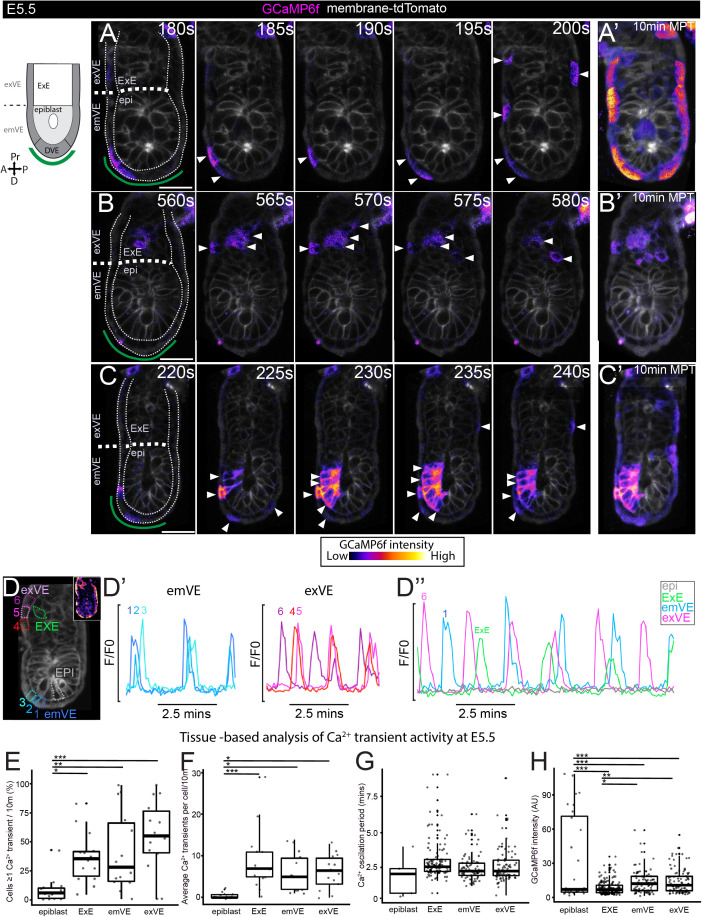
At E5.5 Ca^2^+ transients are more widespread and occur with higher frequency in extraembryonic than embryonic tissues. **(A–C)** Selected time points of three E5.5 GCaMP6f:membrane-tdTomato embryos imaged every 5-s for 10 min showing Ca^2+^ transients in VE, ExE, and epiblast. Green line in first frame indicates approx. position of DVE cells based on morphology—no obvious difference in dynamics could be observed in these cells when compared to the surrounding emVE and exVE. **(A′, B′, C′)** Max-intensity temporal projections (MTP) of full 10-min timelapse (MTPs) (see S10 Movie). **(D)** E5.5 embryo with ROI’s relating to D–D″. **(D′–D**″) Traces of GCaMP6f fluorescence for emVE, exVE showing neighboring cells undergoing Ca^2+^ oscillations at a similar periodicity. **(D**″) The timing of Ca^2+^ transients is different across cells. Epiblast cells are largely quiescent. **(E–H)** E5.5 embryos (*N* = 14) analyzed using a trained CNN peak detector. **(E)** Graph showing percentage of cells in each embryo that showed ≥1 transient. Epiblast has significantly fewer transients than other tissues (one-way ANOVA, *p* ≤ 0.05, followed by Tukey’s HSD Test on epiblast vs: ExE, *p* ≤ 0.05, emVE *p* ≤ 0.01, exVE *p* ≤ 0.001)). **(F)** Graph of average number of Ca^2+^ transients per cell. Epiblast cells show the fewest transients (one-way ANOVA, *p* ≤ 0.001, followed by Tukey’s HSD Test on epiblast vs: ExE, *p* ≤ 0.001, emVE *p* ≤ 0.05, exVE *p* ≤ 0.05)). **(G)** Graph of Ca^2+^ oscillation periodicity for all cells. **(H)** Graph showing GCaMP6f fluorescent intensity at Ca^2+^ transient peaks. Epiblast Ca^2+^ transient peak intensity were on average significantly higher than other tissues (one-way ANOVA, *p* ≤ 0.001, followed by Tukey’s HSD Test on epiblast vs: ExE, *p* ≤ 0.001, emVE *p* ≤ 0.001, exVE *p* ≤ 0.001)). emVE and exVE showed a slightly higher intensity than the ExE (one-way ANOVA, *p* ≤ 0.001, followed by Tukey’s HSD Test on ExE: vs: emVE, *p* ≤ 0.05, exVE *p* ≤ 0.01)). Note that the emVE was not subdivided into DVE and non-DVE for tissue-based analysis. All scale bars = 25 μM. Tabulated data for E–H can be found in S1 Data.

### Kymograph visualization of GCaMP6f Ca^2+^ reporter activity

To visualize GCaMP6f Ca^2+^ reporter activity in timelapses of E5.5 embryos, individual traces were stacked together as a kymograph, each row being the normalized intensity trace, each column a time point, with signal intensity colored using the ‘magma’ color-scheme. The yellower/whiter the color, the stronger the trace intensity. The purpler/blacker the coloring, the weaker the trace intensity. Traces were normalized by min–max normalization, (trace − min)/(max − min) where min and max are the minimum and maximum intensity across all traces within an embryo. The color-scheme was then applied to the value range [0,1]. Traces are row-sorted by performing an indirect stable sort with cluster number first and the time of its first peak second. For global clustering, where individual traces were z-scored per embryo instead of min–max normalized, the same row-sorting is applied but with the kymograph coloring applied to the positive value range [0, 2].

### Spatiotemporal analysis of Ca^2+^ transients in E5.5 embryos

To investigate the spatiotemporal pattern of calcium transients in E5.5 GCaMP6f:membrane-tdTomato timelapses obtained at 5-s interval for 10 min duration, we used Motion Sensing Superpixel (MOSES) tracking [47]. As Ca^2+^ transients occurring sequentially in neighbor cells or from region to region within a cells, will appear to move like a wave, we used super pixel motion tracking to analyze the behavior of the GCaMP6f reporter. We applied MOSES with [48] to extract the mean temporal Ca^2+^ flow pattern for each embryo. To ensure minimal bleed-through of cell movements, a binary threshold of mean + 0.5 standard deviation of the calcium signal was used prior to tracking. To enable comparison across embryos, all embryos were rotated to be vertical with ExE top by applying principal components analysis to the cell centroids. Flow maps showing the average directional motion behavior of the calcium transients were then generated. For flow maps of epiblast waves, we applied MOSES with TV-L1 optical flow [49,50] which is more suitable for capturing large displacements.

### Whole-mount immunofluorescence

Whole-mount immunofluorescence was performed as previously described [10]. Briefly, embryos were fixed in 4% PFA for 20 min at room temperature then washed three times for 5 min each in 0.1% Triton-X100 in phosphate-buffered saline (PBS); incubated in 0.25% Triton-X100 in PBS for 25 min; washed three times in 0.1% Tween-20 in 1×PBS; then blocked with 2.5% donkey serum, 2.5% goat serum, and 3% Bovine Serum Albumin (BSA) in 0.1% Triton-×100 in PBS overnight. Embryos were then incubated at 4 °C overnight in primary antibodies diluted in 1:100 in block. Embryos were washed three times in 0.1% Tween-20 in PBS (PBT) for 5 min each, with a final wash of 15 min; incubated overnight at 4 °C with appropriate secondary antibody 1:100 in 0.1% PBT and 1 nM phalloidin overnight at 4 °C. Finally, embryos were washed four times for 5 min in PBT at room temperature; and mounted with Vectashield mounting media containing 4′,6-diamidino-2-phenylindole (DAPI) (Vector Labs H-1200).

### Antibodies and stains

Primary antibodies used were: rabbit anti-Cleaved Caspase-3 (Cell Signaling, 9961S, Lot: 43 (06/2014)), rabbit anti-Phospho-Histone H3 (S28) (Cell Signaling, 9713P, Lot: 2 (09/2013)), rabbit anti-OCT-4 (Abcam, ab200834, Lot: GR212680−16), rabbit anti-CDX2 (Cell Signalling, 9775, Lot: 3(12/2017)). All primary antibodies were used at 1:100 dilution. Secondary antibodies used were: donkey anti-rabbit Alexa Fluor 555 (Invitrogen, A31570, Lot: 1945911). For F-actin staining phalloidin-Atto 647N (Sigma, 65906) was used at a 1 nM final concentration in 1 × PBT. Nuclei were stained by Vectashield mounting media containing DAPI (Vector Labs H-1200).

### Confocal microscopy of fixed samples

Fixed E5.5 embryos were imaged following the immunofluorescence protocol (as above) on a LSM 880 (ZEISS) confocal microscope using a 40× oil (1.36NA) objective. Z-stacks of embryos were acquired at 1 μm interval using non-saturating parameters. Max intensity projections (MIPs) were made using ZEN black software (ZEISS). Figures were prepared with Adobe Photoshop and Adobe Illustrator (Adobe).

### Analysis of apoptosis in thapsigargin-treated embryos

For analysis of apoptosis, the level of Cleaved Caspse-3 (Casp3) was compared between thapsigargin-treated and DMSO as a solvent control. 3D z-stacks of cultured embryos stained for F-actin and Casp3 were used to analyzed the percentage of staining in each embryo as follows: first the F-actin channel was used to manually segment the 3D volume of each embryo using FIJI. The Casp3 channel was then automatically segmented using FIJI Particle Analyzer plugin, enabling the percentage of Casp3 staining in each embryo to be calculated. Statistical tests were carried out using R (ver 1.1.45).

## Results

### Ca^2+^ transients in the pre-implantation mouse embryo are rare stochastic events

In order to image cytosolic Ca^2+^ transients in all tissues of the early mouse embryo, we generated a constitutively and ubiquitously expressed version of the GCaMP6f Ca^2+^ reporter line [39], henceforth referred to as “GCaMP6f” (see Methods). We crossed this line with *Gt(ROSA)26Sor*^*tm4(ACTB-tdTomato,-GFP)Luo*^ *“*mT/mG” mice [41] to generate embryos in which cells and tissues could be discerned with a fluorescent membrane-tdTomato signal while recording cytosolic Ca^2+^ dynamics with the GCaMP6f reporter. To investigate whether cytosolic Ca^2+^ transients occur at early developmental stages, we performed a stage-series of high-time resolution confocal timelapse imaging of freshly dissected embryos from a range of stages (E0.5, E1.5, E2.5, E.3.0, E3.5, E4.5 and E5.5). As Ca^2+^ levels could change slowly or rapidly, in order to minimize photo-toxicity we employed an imaging strategy to accommodate these possibilities using a combination of high time-resolution short-duration (typically 5 second intervals for 10 min), and lower time-resolution longer-duration imaging parameters (for example 20 min intervals for 10 hours) varying with the stage (see Methods and S1 Table).

We defined a Ca^2+^ transient as a cytosolic increase in GCaMP6f fluorescence, followed by return to basal levels. If a cell showed more than one Ca^2+^ transient within our imaging duration, we termed this a Ca^2+^ oscillation. If Ca^2+^ transients occurred sequentially across neighboring cells in a directional manner, we termed this a wave.

At E0.5, we imaged GCaMP6f:membrane-tdTomato embryos from post-pro-nucleus formation until the 2-cell stage (*N* = 5) (S1 Movie). No Ca^2+^ transients were observed in any embryo (*N* = 5). Next, we imaged E1.5 embryos (*N* = 13) from the 2-cell until the 4-cell stage (S2 Movie). Though we did not observe any large changes in cytosolic Ca^2+^ concentration, upon division from 2 to 4 blastomeres, a higher relative level of Ca^2+^ appeared along the interface between dividing cells (S2 Movie) similar to what has been shown for other species at these stages of development [51–53]. We also noted that there were multiple punctate regions within the cytosol with a higher relative level of Ca^2+^ (Fig 1A–1A′ and S3 Movie) that appear stable over short-term imaging (5-s interval for 10 min) (S4 Movie). At E2.5, the punctate regions of high GCaMP6f signal were now less obvious around the nuclei, but seemed to be more prevalent near the apical (outer) surface of blastomeres (Fig 1B–1B′ and S5 Movie). At E3.0, we could still observe cytosolic clusters of puncta similar to those at E2.5 (*N* = 7) (Fig 1C–1C′ and S6 Movie) and in one embryo, we also observed three blastomeres with a single Ca^2+^ transient occurring in our 10 min imaging duration.

Interestingly at E3.5, all embryos (*N* = 6) showed higher levels of Ca^2+^ in trophectoderm cells relative to the ICM (Fig 1D–1D′ and S7 Movie). A single Ca^2+^ transient was also observed in a trophectoderm cell in one embryo (S7 Movie). At E4.5, trophectoderm and primitive endoderm cells showed an elevated level of Ca^2+^ compared to the pluripotent epiblast (*N* = 5) (Fig 1E–1E′ and S8 Movie), but no Ca^2+^ transients were observed. Together, these findings suggest that between E0.5 and E4.5 cytosolic Ca^2+^ transients are rare events. At early stages, small cellular vesicles with Ca^2+^ levels higher than the surrounding cytosol are localized either around the nucleus or near the apical domain. From blastocyst stages, extraembryonic tissues show a relatively higher cytosolic Ca^2+^ level than the pluripotent ICM (E3.5) or epiblast (E4.5).

### E5.5 marks the onset of widespread periodic Ca^2+^ transients

At E5.5, following implantation, the embryo elongates to form the egg-cylinder, with the epiblast abutting the proximally located extraembryonic ectoderm (ExE), both surrounded by the monolayer epithelium of the VE (termed emVE when overlying the epiblast, and exVE overlying the ExE) (Fig 2A, see diagram). Strikingly, when imaged at 5-second intervals, we observe periodic Ca^2+^ transients throughout the emVE, exVE (Fig 2A–2A′ and S9 and S10 Movies), as well as in the ExE (Fig 2B–2B′ and S10 Movie, top right), with several cells undergoing multiple transients (Ca^2+^ oscillations) within a 10 min imaging period (N = 14). Transients were not temporally coordinated across the embryo, but we noted that they often occurred sequentially in small groups of neighboring cells in the emVE (Fig 2D–2D′), exVE (Fig 2D–2D′) and ExE (Fig 2B and 2D″ and S10 Movie). At this stage a sub-set of VE cells, the DVE, are induced at the distal tip of the egg cylinder and become more columnar than surrounding cells (Fig 2A–2C, green region). Though we could observe Ca^2+^ oscillations in these cells, they did not appear to show Ca^2+^ behavior distinct from surrounding emVE cells (Fig 2A–2C, see green region). To test whether there was any spatial bias to these events, we applied MOSES [47] analysis (see Methods) to show flow patterns of transients across the ExE and VE (S3 Fig). This revealed that there was no directional bias in the transients, which could move either distally (see asterisk in S3A˝ Fig) or proximally (see asterix in S3B˝ Fig) within distinct groups of cells. We also imaged E5.5 embryos still attached to maternal tissue (decidual endometrium), and prior to the removal of the extraembryonic Reichert’s membrane showing that widespread calcium oscillations exist prior to complete removal of the embryos from their uterine environment (*N* = 3 embryos) (S11 Movie).

As this analysis was based on single-optical sections, where a wave traveling out of the imaging plane would be missed, we next captured 3D volumes of E5.5 embryos at 10-s intervals using a Z.1. lightsheet microscope (S12 Movie). This confirmed that though there are Ca^2+^ transients across the VE and ExE, there are no larger wave-like events across these tissues (*N* = 3). Together, these suggest local interconnectivity between neighboring cells exists, but there is no directional bias.

At this stage, Ca^2+^ transients could also be observed in the epiblast, though much less frequently (Fig 2C and S10 Movie, bottom left). In contrast to the VE and ExE, epiblast transients spread progressively across multiple cells in a wave-like manner and appeared to have a higher intensity (Fig 2C and 2D′″ and S10 Movie). Unlike at earlier stages (E1.5–E3.0) no vesicles with a higher level of Ca^2+^ were observed in any cells at E5.5 embryo.

In order to characterize the number, duration, and location of Ca^2+^ transients in more detail, we used the membrane-tdTomato signal and manually outlined regions of interest (ROI) around each cell to create a dataset of 1,126 cells (323 epiblast, 430 ExE, 205 emVE, and 168 exVE from all the E5.5 embryos (*N* = 14) imaged at 5-s interval for 10 min. As we lacked a DVE marker, we did not subdivide the emVE into the DVE for this analysis. We manually annotated the peaks of Ca^2+^ transients from three embryos and used this to train a CNN “Ca^2+^ peak detector” (see Methods). We then applied the trained CNN Ca^2+^ peak detector to identify transients in the remaining embryos and analyzed at the tissue level: the proportion of cells in each tissue that showed at least one Ca^2+^ transient; the number of Ca^2+^ transients in each cell; the average time between Ca^2+^ transients in a cell (oscillation period); the duration of Ca^2+^ transients and; the relative level of GCaMPf fluorescence at Ca^2+^ transient peaks.

We found that the proportion of cells with ≥1 Ca^2+^ transient at E5.5 was significantly higher in all extraembryonic tissues (ExE, emVE, exVE) than the epiblast (Fig 2E). Only 10% (32/323) of epiblast cells imaged showed ≥1 Ca^2+^ transient compared to 38% (163/430) of ExE, 40% (83/205) of emVE, and 56% (94/168) of exVE cells (Fig 2E). The average number of transients per cell was also significantly higher in extraembryonic (~5–8 transients in 10 min) than epiblast cells (~1 transient in 10 min) (Fig 2F and S1 Data). The duration of Ca^2+^ transients (see Methods) was similar across tissues at around 20 s (S1 Data). The average Ca^2+^ oscillation period was similar between cells in extraembryonic tissues (~3 min), but cells also showed a wide range of oscillation periods (30 s to 9 min) (Fig 2G and S1 Data). While the average oscillation period for epiblast was shorter (~2 min) this only represented a total of 5 events across the entire dataset, since an oscillation in the epiblast was a rare event (Fig 2G and S1 Data). Lastly, we analyzed the relative intensity of Ca^2+^ transients. This revealed that epiblast had a greater range cytosolic Ca^2+^ levels compared to the extraembryonic tissues (Fig 2H and S1 Data).

### Unbiased clustering of E5.5 cells reveals different cells types with distinct Ca^2+^ handling behaviors

**Fig 3:**
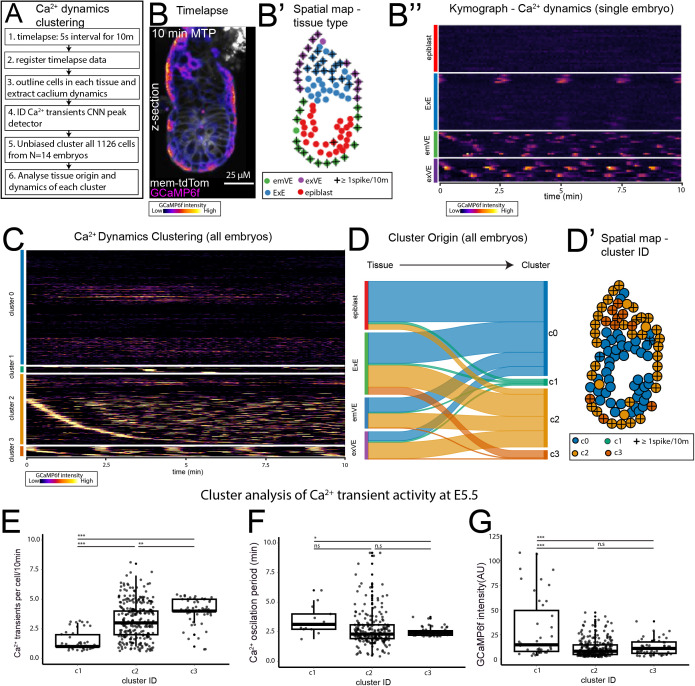
Unbiased clustering of Ca^2^^+^ dynamics reveals four activity types. **(A)** To assess the similarity of Ca^2+^ transients in all cells from E5.5 (*N* = 14) embryos imaged at a 5-s interval for 10 min unbiased clustering of Ca^2+^ transients using a hierarchical density-based clustering was performed. **(B)** Example max-intensity time projection of an example E5.5 GCaMP6f embryo. **(B′)** Spatial map of cell-type and Ca^2+^ activity on embryo in **B. (B**″**)** Kymograph of E5.5 embryo in B, showing GCaMP6f activity during 10 min. **(C)** Kymograph of GCaMP6f activity of 1,126 cells from *N* = 14, E5.5 embryos clustered using hierarchical density-based clustering. Four clusters were identified with cluster 0 representing little-no activity. **(D)** Sankey graph showing contribution of cells from each tissue to the four cluster types. Epiblast contribute predominantly to c0 (no spike), while extraembryonic cells contribute to all clusters, more cells from ExE contribute to c3, while the majority of the emVE and exVE contribute to c2. **(D′)** Spatial map of embryo in B, showing cluster ID. **(E–F)**. Quantification of GCaMP6f activity in clusters 1–3. **(E)** Cluster1 had the lowest number of Ca^2+^ transients, while cluster 3 had the highest (one-way ANOVA, *p* ≤ 0.05, followed by Tukey’s HSD Test on c1 vs. c2, *p* ≤ 0.001, c3. c2 vs. c3:0.01). **(F)** Ca^2+^ oscillation period showed that cluster 1 had slower oscillations than cluster 3 (though c1 data reflected a total of only 13 cells), while there was no signifiant difference between clusters 2 and 3 (one-way ANOVA, *p* ≤ 0.05, followed by Tukey’s HSD Test on c1 vs. c3, *p* ≤ 0.05, c2 vs. c3, *p* ≥ 0.05). **(G)** Intensity of Ca^2+^ transients (AU, arbitrary unit) in each cluster. Cluster 1 transients had on average significantly higher intensity (one-way ANOVA, *p* ≤ 0.05, followed by Tukey’s HSD Test on c1 vs. c2, *p* ≤ 0.001, c3, *p* ≤ 0.001). Tabulated data for E–G can be found in S2 Data.

While these analyses enable comparison of the average behavior of cells in each tissue, they do not take into account the spatio-temporal pattern of behavior. We therefore performed an unbiased clustering based analysis of our data using HDBSCAN [46], a hierarchical density clustering approach (Figs 3A–3B′ and S1 and see Methods). We also plotted Ca^2+^ dynamics in each embryo as a kymograph to visualize the temporal behavior of cells across all tissues during 10-min timelapse experiments to be easily visualized (Figs 3B″ and S2 and see Methods). We clustered all cells (*N* = 1,126) from our E5.5 timelapse dataset (*N* = 14 embryos) according to the similarity of their Ca^2+^ dynamics and plotted this as an integrated kymograph (Fig 3C). We found that cells clustered into four groups (clusters 0, 1, 2, 3), with cluster 0 representing cells with no or trace Ca^2+^ transient activity (Fig 3C and S2 Data) over the 10-min duration of imaging. We then analyzed the contribution of cells from tissues to each cluster (Fig 3D) and plotted this onto spatial maps for each embryo (Figs 3D′ and S2). This showed that while the epiblast is made up predominantly of cells from c0 (no transient) (Fig 3D′), extraembryonic tissues are made up of cells from all clusters (Fig 3D′). Cluster c3 is made up predominantly of ExE, while c2 is made up predominantly of ExE, emVE, and exVE (Fig 3D′).

To differentiate the behavior of Ca^2+^ transients between the clusters that showed at least one transient (clusters 1–3) we analyzed the number of Ca^2+^ transients, the oscillation periodicity, and intensity of Ca^2+^ transients in each cluster (Fig 3E–3G and S2 Data). This showed that cluster 1–3 represent a gradient of lower to higher activity with cluster 1 cells showing fewer transients with the majority of cells showing only 1 transient in 10 min (Fig 3E and S2 Data), a similar oscillation periodicity (Fig 3F and S2 Data) but at a higher intensity (Fig 3G) than other clusters, while clusters 2 and 3 show higher numbers of transients, but a similar oscillation periodicity and transient intensity (Fig 3E–3G and S2 Data). Together, these results show that cells in the E5.5 embryo can be categorized into different groups based on Ca^2+^ dynamics and that these groups broadly correspond to tissue differences. While epiblast cells are largely confined to either no transients (cluster 0), or infrequent high intensity transients (cluster1), the extraembryonic tissues largely cluster to higher frequency, average intensity transients (cluster 2–3).

### Ca^2+^ transients in epiblast cells propagate from the apical to basal domain

**Fig 4:**
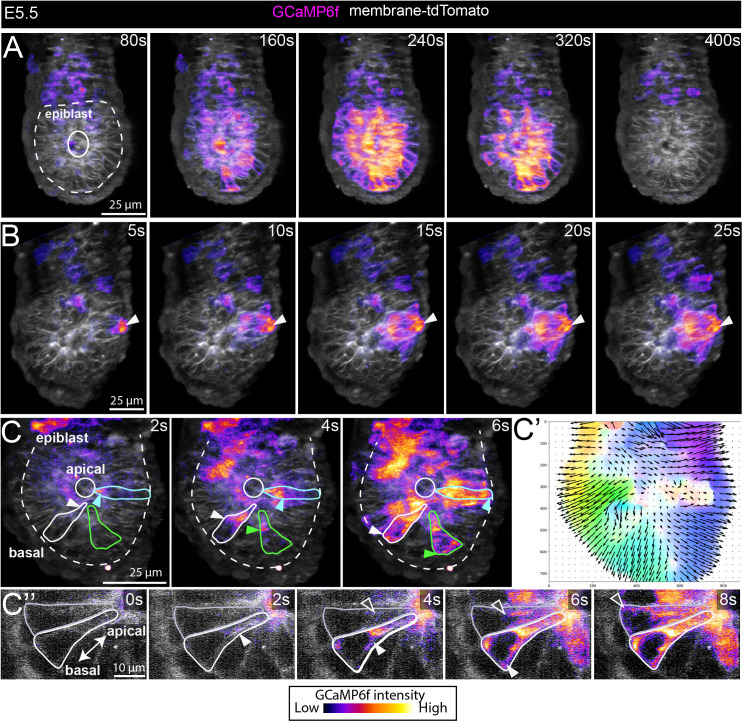
Epiblast Ca^2^+ waves propagate from the apical to basal domain. **(A–C**″**)** Selected time points from movies of E5.5 GCaMP6f embryos imaged using a ZEISS lattice light-sheet 7 microscope. **(A)** An example of an epiblast wave involving a large proportion of the tissue captured by imaging a volume of the embryo at 20-s intervals (also see S13 Movie). A total of 9/16 embryos showed at least one epiblast Ca^2+^ wave. **(B)** Faster imaging at 5-s interval revealed that the Ca^2+^ wave in the epiblast can be preceded by a transient in a neighboring VE cell—this was observed in 5/19 events from 9 embryos (white arrowheads) (S14 Movie). **(C)** Imaging at 2-s interval captured the intracellular propagation of the Ca^2+^ transient; Ca^2+^ activity begins at the apical domain of epiblast cells and moves toward the basal aspect of cells (white-, cyan-, and green-arrowheads denote the position of Ca^2+^ activity in 3 outlined epiblast cells) (*N* = 3 embryos) (see S15 Movie). **(C′)** Unbiased super-pixel motion analysis showing the intracellular apical to basal movement in the embryo shown in **C. (C**″**)** Selected time points from a single optical section from the embryo in C showing the apical-basal movement of Ca^2+^ activity in 2 outlined epiblast cells. All images are maximum intensity projections.

While Ca^2+^ transients in the epiblast were rare in a single optical section imaged over a 10-min duration (Fig 3C and 3D), when observed, these events were expansive, propagating across multiple cells as a wave (e.g., Fig 2C). To investigate this phenomenon in more detail and across the 3-dimensional extent of the embryo, we imaged GCaMP6f:mTmG embryos using LLSM, which enabled us to capture events in the near-full volume of the E5.5 embryo and over a longer imaging duration (5 or 20 s interval between volumes over 20 min, or 20 s interval for 5 h, see S1 Table). This confirmed our previous observations that epiblast Ca^2+^ transients occurred in a wave-like manner, sequentially propagating across neighboring cells of the epiblast. Moreover, volume imaging showed that there is variability in the extent of the epiblast across which the wave traveled. In some cases, covering the majority of epiblast cells (Fig 4A and S13 Movie), while in other cases, it was more limited in extent (Fig 4B and S14 Movie). We observed a single epiblast wave in 6/13 embryos imaged for 20 min (3/10 in embryos imaged at 20-s intervals and 3/3 in those imaged at 5-s intervals). When imaged over a longer, 5-h duration, 3/3 embryos showed multiple epiblast waves; 2 embryos had 4 waves, 1 embryo had 3 waves. These waves lasted for an average of 70 s  ± 37 s, but could be as short as 20 s. The time between epiblast waves was on average 25 min ±16 min, with a maximum interval of 57 min. The average speed of transients in an individual epiblast cell was 15.41 μm/s ± 9.49 (mean ± SD; *n* = 6 epiblast cells).

In 5/19 waves from the 9 embryos that showed at least one wave, we also noted that the initiation of the epiblast Ca^2+^ wave was preceded by a transient in a VE cell immediately adjacent to the origin of the epiblast wave (Fig 4B and S14 Movie). The Ca^2+^ transient in these VE cells were of high intensity and persisted for longer than the transients in the epiblast cells, returning to resting levels only after the epiblast wave had ended (Fig 4B and S14 Movie). Due to the imaging interval and the fact that we are unable to capture the full embryo volume, it is unclear whether a Ca^2+^ transient in a VE cell always proceeds an epiblast wave, or if this represents only a proportion of these events.

To understand the intracellular propagation of the Ca^2+^ transient within individual cells, we imaged embryo volumes at a higher temporal resolution, at 2-s intervals using LLSM. This revealed that Ca^2+^ transients originate from the apical aspect of epiblast cells and moves toward their basal aspect (all epiblast cells in *N* = 3 embryos) (Fig 4C and S15 Movie). We confirmed this in an unbiased manner by applying MOSES analysis [47] to track the Ca^2+^ signal as pixel motion (Fig 4C′).

### Ca^2+^ transients in the E5.5 embryo are dependent on SERCA activity

**Fig 5:**
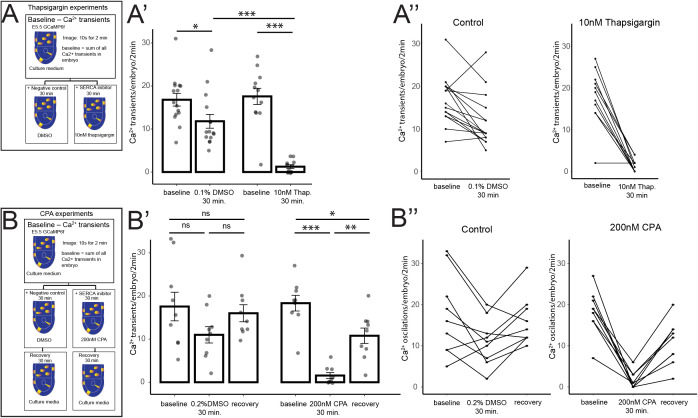
SERCA is required for Ca^2^+ transients at E5.5. To test the requirement of endoplasmic reticulum (ER) for Ca^2+^ dynamics at E5.5, GCaMP6f embryos were treated with SERCA inhibitors, and the total number of Ca^2+^ transients measured. **(A)** Diagram of experimental method for nonreversible SERCA inhibitor thapsigargin. Embryos were imaged at a 10-s interval for 2 min. **(A′)** Graph of Ca^2+^ transients in embryos treated with 0.1% DMSO and 10 nM thapsigargin. DMSO treated embryos had a significant decrease in transients (one-way ANOVA, *p* ≤ 0.001, followed by Tukey’s HSD Test; baseline vs. 30 min DMSO *p* > 0.05), but thapsigargin almost completely abolished transients to a significant effect (one-way ANOVA, *p* ≤ 0.001, followed by Tukey’s HSD Test, baseline vs. 30 min 10 nM thapsigargin *p* ≤ 0.001), that was significantly lower than DMSO controls (one-way ANOVA, *p* ≤ 0.001, followed by Tukey’s HSD Test, 30 min DMSO vs. 30 min 10 nM thapsigargin: *p* ≤ 0.001). **(A**″**)** Graphs of individual embryos from A′, traced from baseline to post-30-min culture. **(B)** Diagram of experimental method for the reversible SERCA inhibitor cyclopiazonic acid (CPA). **(B′)** Graph of Ca^2+^ transients in DMSO and 200 nM CPA treatment. While 0.2% DMSO-treated embryos showed a decrease in transients, it was not significant (one-way ANOVA, *p* ≤ 0.001, followed by Tukey’s HSD Test, baseline vs. 30 min 10 nM thapsigargin: *p* ≥ 0.05). 200 nM CPA treatment almost completely abolished the Ca^2+^ transients to a significant effect (one-way ANOVA, *p* ≤ 0.001, followed by Tukey’s HSD Test, baseline vs. 30 min 200 nM CPA: *p* ≤ 0.001), but embryos were able to recover significantly after CPA was washed off and cultured with fresh medium (one-way ANOVA, p=<0.001, followed by Tukey’s HSD Test, 30 min 200 nM CPA vs. recovery: *p* ≤ 0.01). **(B**″**)** Graphs of individual embryos from B′, traced throughout the experiment. Tabulated data for A–B″ can be found in S3 Data.

Having identified the onset of cytosolic Ca^2+^ transients at E5.5 we next wanted to understand the source of Ca^2+^ these may represent. The ER is a key intracellular Ca^2+^ store. After the release of Ca^2+^ from these stores into the cytosol, the SERCA transport Ca^2+^ from the cytosol back into the ER [16] to replenish stores in readiness for the next transient. To determine if the transients we observed were dependent on this mechanism, we cultured GCaMP6f embryos in thapsigargin, a cell-permeable SERCA inhibitor [54,55] widely used in developmental studies [29–31,56–58]. We assessed the total number of Ca^2+^ transients in embryos immediately post-dissection (‘baseline’), and again after 30 min of culture with 10 nM thapsigargin, or DMSO as a negative control (Fig 5A). Though control embryos cultured in DMSO did show a statistically significant decrease in the number of transients (from an average of 17 at baseline, to 12 Ca^2+^ transients after culture, *N* = 12 embryos, S3 Data), treatment with 10 nM thapsigargin resulted in a near complete abolition of Ca^2+^ transients (from an average of 18, at baseline, to 1.3 Ca^2+^ transients postculture, *N* = 12 embryos, S3 Data). Of these, 6/12 had a complete loss of Ca^2+^ transients (Fig 5A–5A″ and 5B and S3 Data).

To further test whether SERCA is required for cytosolic Ca^2+^ activity at E5.5, we treated embryos with a second inhibitor; CPA [59], also previously used in developmental studies [30,31,57]. Like thapsigargin, CPA inhibits SERCA but has the advantage that it acts in a reversible manner [59]. We therefore assessed the number of Ca^2+^ transients in E5.5 embryos immediately post-dissection (“baseline”), after 30 min culture with 200 nM CPA, or DMSO as a solvent control, and then assessed embryos ability to recover after culturing in fresh medium for an additional 30 min (Fig 5C). While DMSO control embryos showed a similar reduction in the number of Ca^2+^ transients as before, though in this case not statistically significant (Fig 5C–5C″ and S3 Data), CPA almost completely abolished all Ca^2+^ transients after 30 min culture (from an average of 18 to 1.6 Ca^2+^ transients post-culture, *N* = 9 embryos). Of these, 4/9 showed a complete loss of Ca^2+^ transients (Fig 5C–5C″ and 5D and S3 Data). CPA-treated embryos were able to recover significantly, to approximately half of their initial baseline level after CPA was washed off (Fig 5C–5C″ and 5D and S3 Data). Together, these data indicate that Ca^2+^ transient activity at E5.5 is SERCA-dependent.

### Ca^2+^ signaling is required for DVE migration

**Fig 6:**
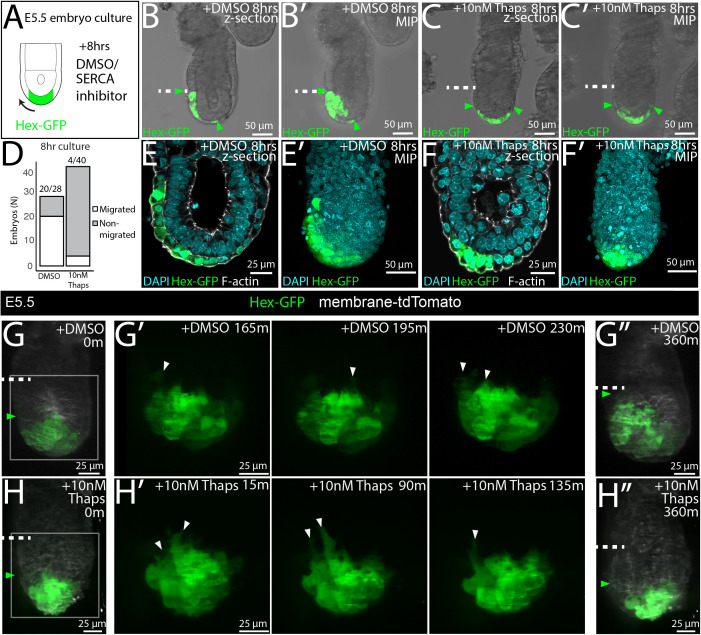
Inhibition of SERCA arrests DVE migration at E5.5, but does not block DVE cell projections. **(A)** Diagram of experimental method showing the culture of Hex-GFP embryos with 10 nM thapsigargin or 0.1% DMSO or as a control. **(B–B′)** Example E5.5 embryo showing a single z-section and max-intensity projection (MIP) after 8 h culture with DMSO (B–B′), or 10 nM thapsigargin **(C–C′)** (green arrows mark position of the DVE, white dashed-line; emVE-exVE boundary). **(D)** Graph showing the number of embryos that migrated in DMSO, or 10 nM thapsigargin treatment after 8 h. While in 71% (20/28) of DMSO controls embryos the DVE migrated, only 10% (4/40) of thapsigargin-treated embryos were able to migrate. **(E–F′)** Example Hex-GFP embryos post-fixation after 8-h culture showing DMSO and 10 nM thapsigargin-treated embryos stained with DAPI and Phalloidin (F-actin) showing that VE cells remain as an intact monolayer in both control and thapsigargin-treated embryos. **(G, H)** Example of E5.5 embryos imaged at 5 min intervals for 6 h using a Lattice Lightsheet microscope while being cultured in DMSO **(G–G**″**),** or 10 nM thapsigargin **(H–H**″**)** (green arrows mark the proximal position of DVE cells, white dashed line; emVE-exVE boundary). H′ and G′ show the Hex-GFP channel in the boxed region in H and G, for 3 selected time points during the timelapse to highlight anteriorwards cellular projections (white arrowheads) were present in both culture conditions. G″ and H″ show the position of the DVE at the end of the 6-h culture imaging period. While embryos imaged in both DMSO and thapsigargin showed cellular projections, only 7.7% (2/26) embryos culture with thapsigargin migrated in the 6 h culture compared to 76% (13/17) of DMSO controls (see S16 Movie).

At E5.5, the DVE cell population undergoes a cell migration within the VE epithelium [2]. As Ca^2+^ signaling has been shown to regulate cell migration in a range of cellular contexts [19–21], and given the wide-spread Ca^2+^ activity in this tissue, we asked whether it is required for the migration of DVE cells. To test whether Ca^2+^ activity is required for DVE migration, we cultured E5.5 Hex-GFP embryos, where DVE cells are marked by cytoplasmic EGFP, with 10 nM thapsigargin or DMSO as a solvent control, and assessed the extent of their migration after 8 hours (Fig 6A). While 71% (*N* = 20/28) control embryos showed DVE migration (Fig 6A–6A′ and 6D), in only 10% (*N* = 4/40) of thapsigargin-treated embryos did the DVE migrate (Fig 6C–6C′ and 6D, also see S4A–S4B Fig), with Hex-GFPve cells instead arrested at the distal tip of embryos (Figs 6E–6F′ and S4A′). We found a similar inhibition of DVE migration when embryos were cultured in 200 nM CPA (only 6/25 embryos showed DVE migration when cultured for 10 h with 200 nM CPA compared with 14/14 control embryos), suggesting that SERCA regulation of Ca^2+^ activity is required for DVE migration.

As Ca^2+^ signaling can also regulate a wide range of cellular processes, including proliferation, apoptosis, and cell fate, we fixed embryos that had been cultured in 10 nM thapsigargin or DMSO for 8 hand performed whole-mount immunohistochemistry for key markers. We found no significant difference in the number of cell divisions as marked by phospho-Histone H3 (S4C–S4C′ Fig and S4 Data), nor in the apoptotic marker Cleaved Caspase-3 (S4D–S4D′ Fig and S4 Data). Though there was no effect on apoptosis or cell proliferation, thapsigargin-treated embryos were on average 7% smaller than DMSO cultured controls (S4E Fig and S4 Data). Finally, we found no difference in key cell identity markers of the epiblast (OCT4) (S5A Fig) or extraembryonic ectoderm (CDX2) (S5B Fig), nor to the distribution of F-actin (S5C–S5C′ Fig).

### Cellular projections in DVE cells are not inhibited by thapsigargin-treatment

We next investigated the cellular mechanism underpinning thapsigargin-mediated DVE arrest. As Ca^2+^ signaling has been shown to regulate cell polarity and filopodial projections in migrating cells [19,20,60] and DVE cells generate basally-located projections polarized in the direction of their migration [2,11], we investigated whether thapsigargin blocked DVE cellular projections. We imaged E5.5 Hex-GFP embryos treated with 10 nM thapsigargin or DMSO as a solvent control, using high-resolution LLSM to capture entire image volumes every 5 min over the course of 6 h. In 76% (*N* = 13/17) of control embryos, the DVE successfully migrated within 6 h, and DVE cells in these embryos showed basal projections (*N* = 17/17; Fig 6G–6G″ and S16 Movie). Thapsigargin-treated embryos showed a failure to migrate with only 8% (*N* = 2/26) migrating within 6 h, but imaging revealed that DVE cells in all embryos retained dynamic cellular projections (*N* = 26/26; Fig 6H–6H″ and S16 Movie). This indicates that SERCA inhibition of DVE migration is not due to the loss of basal projections in DVE cells.

## Discussion

Ca^2+^ signaling can control a diverse range of cellular processes [12–14], but its potential role during early mouse development has not been well characterized. Using a transgenic Ca^2+^ reporter mouse, we performed a detailed assessment of Ca^2+^ activity between E0.5 and E5.5 by live imaging. We find that Ca^2+^ transients, though initially rare events, become upregulated in a tissue-specific manner as development progresses. Extraembryonic tissues such as the E5.5 VE and ExE show increased occurrences of Ca^2+^ transients but, in contrast, the pluripotent epiblast remains relatively quiescent, with only occasional, large-scale Ca^2+^ waves that spread across the tissue. We show that the Ca^2+^ transients in the VE are functionally important, since inhibition of transients by blocking SERCA leads to migration arrest of the DVE cell population that is required for correct A-P patterning.

Ca^2+^ has been show to control several aspects of cell migration in other contexts including; the front-back polarity of cells [19,20], the behavior of leading cells [21], and the regulation of lamellipodia [60]. However, in this study, we find that DVE cells do not show any differences in cytosolic Ca^2+^ level relative to surrounding VE cells, nor a clear front-to-back gradient within cells. Furthermore, we find that DVE cells retain dynamic cellular projections when cultured with inhibitors of SERCA. This indicates that such projections are either not sufficient to drive migration, or those that are present are somehow qualitatively altered so that they no longer can facilitate DVE migration, for example, by affecting the regulation of focal adhesions [61,62]. Alternatively, the role of Ca^2+^ could be non-cell autonomous—DVE cells must migrate through the emVE epithelium, with surrounding cells undergoing cell–cell rearrangements to accommodate the migrating cells [10,63]. As Ca^2+^ activity occurs throughout the VE, it could regulate cell–cell interactions, for example, via the WNT-PCP pathway [29], which has also been implicated in DVE migration [10]. Alternatively, it could act through modulating the turn-over of cell–cell adhesion molecules, for example, E-cadherin that is expressed throughout the DVE and surrounding VE [10], and whose binding is calcium dependent [64]. Additional experiments will be required to establish whether DVE migration arrest in the VE epithelium is cell autonomous or noncell autonomous. Interestingly, control embryos cultured in DMSO also show a decrease in the average number of Ca^2+^ transients, but show no detectible defect in DVE migration. The reduction in transients in the DMSO controls is an order of magnitude lower than that seen upon SERCA inhibition, which suggests that there is a threshold in Ca^2+^ transient activity below which DVE migration is impaired. Furthermore, we note that E5.5 thapsigargin-treated embryos show small difference in embryo size, but no significant difference in the number of dividing cells. As the egg-cylinder embryo undergoes a morphological change as the DVE migrates [65,66], changing the distal half from a roughly spherical shape to an oblate spheroid, in the absence of migration, these associated global shape changes may be absent. This may account for the small difference in embryo size we observe in treated embryos.

In addition to the VE, we also find periodic Ca^2+^ transients in the extraembryonic ectoderm, which serves as an important developmental signaling center [3,7]. Ca^2+^ has been shown in other contexts to regulate cell fate as well as signaling pathways involved in embryonic development [12–14,58]. Though we find no loss of the key cell fate marker CDX2 in the ExE upon inhibition of Ca^2+^ transients at E5.5, it is currently unclear whether any signaling pathways are affected in this tissue.

Though periodic single-cell Ca^2+^ transients occur throughout extraembryonic tissues at E5.5, we find that they rarely occur in the epiblast. When Ca^2+^ transients are observed in the epiblast, in contrast to those observed in extraembryonic tissues, they span multiple cells and progress from cell to cell as a wave. The difference in Ca^2+^ transient activity between embryonic and extraembryonic tissues is similar to that seen in other species such as *Xenopus* [37], Zebrafish [67], and *Drosophila* [68], and suggests a possible conservation in Ca^2+^ handling across species with respect to the relative quiescence of pluripotent embryonic cells. The intracellular speed of calcium waves in epiblast cells in this study was on the order of 15 μm/s, which is faster than the calcium waves at the furrow of Xenopus embryonic blastomeres (~3–4 μm/s) [53], but slower than the fertilization wave in mouse ooctyes (~31 μm/s, [11], or in the visual system (mouse day 17 retina; ~125 μm/s, [69]. The Ca^2+^ propagation speeds we observe in epiblast cells is consistent with calcium-induced calcium release [70]. Where the epiblast shows a wave of Ca^2+^ transients, we observed in a third of cases that it was preceded by a transient in a neighboring emVE cell, suggesting that the VE cell could be acting as a ‘trigger’ to this event. Whether this is indeed the case will be important to clarify in future experiments, particularly given VE cells are in contact with the basal aspect of epiblast cells, while transients within epiblast cells propagate in an apical to basal direction.

At pre-implantation stages, we observed numerous puncta with high levels of GCAMP6f signal. These are similar to the vesicles (MARVs) and mitochondria observed in previous studies using the Fluo-4 AM dye [28,71], though we do not confirm their identity in this study. We also find that such puncta are not present at later stages when the cytosolic Ca^2+^ transients begin, suggesting that there is a transition in Ca^2+^ handling as development proceeds.

It would be interesting to understand whether there are any differences in calcium activity in embryos developing in utero and in vitro, for example, whether there is any interaction between the maternal site of implantation and the embryo, mediated through reciprocal calcium signaling. However, due to the thickness of the uterine wall and the size of the decidua that envelops the embryo, it is not currently possible to image calcium activity in utero at these early developmental stages.

In conclusion, we have found a novel requirement for Ca^2+^ signaling during anterior–posterior axis formation in E5.5, the mouse embryo. Future work, to understand its mechanism of action in the DVE, and other tissues at this stage will be required using targeted approaches to affect Ca^2+^ signaling in specific cell types.

## Supporting information

S1 Fig Work-flow for unbiased clustering analysis of Ca^2^+ transients at E5.5. **(1)** E5.5 GCaMP6f:membrane-tdTomato embryos (*N* = 14) were imaged at a single z-plane for 5-s interval for 10 min. **(2)** Each cell was manually segmented and given an ID number. Average pixel intensity for each cell was extracted at every time point. **(3)** GCaMP6f intensity for each embryo was plotted as a kymograph with cells ordered by tissue along the y-axis and 5-s interval time points along the x-axis. **(4).** Traces of each cell were pairwise compared to construct a similarity matrix where similarity was defined as the largest positive value of the signal cross-correlation. A hierarchical density-based clustering method, HDBSCAN [46] was then applied to automatically generate clusters. Kymographs were re-plotted to reflect the unbiased clustering and a spatial map of the embryo was colored according to the clusters—showing here clustering results of a single embryo. **(5)** A Sankey plot was generated for each embryo to show the contribution of cells from tissues to each cluster.
(TIF)

S2 Fig E5.5 Ca^2^+ transient clustering. E5.5 GCaMP6f:membrane-tdTomato embryos imaged at a single z-plane for 5-s interval for 10 min. First column shows max-intensity time projection (MTP) of 10 min timelapse. For each embryo, a kymograph and spatial map are shown ordered first by tissue type, then after global clustering. In the final column a Sankey graph shows the contribution of cells in each tissue to the clusters.
(TIF)

S3 Fig Super-pixel motion analysis shows intercellular calcium flows between adjacent cell, but no directional bias at E5.5. **(A–C)** Selected examples of E5.5 GCaMP6f:membrane-tdTomato embryos imaged every 5 s for 10 min. **(A′, B′, C′)** Motion Ca^2+^ transient flow using super pixel tracking. Red arrows represent the local directional motion of the superpixel during 10 min imaging duration. **(A**″**, B**″**, C**″**)** Ca^2+^ transient motion tracking outputs color-coded by vector direction, showing intercellular Ca^2+^ transients can move proximally or distally along the VE.
(TIF)

S4 Fig Analysis of thapsigargin cultured E5.5 embryos. **(A)** Hex-GFP embryos were cultured for 8 h in the SERCA inhibitor thapsigargin. **(A**′**–A**″**)** Additional examples of Hex-GFP embryos showing a single z-section (e1–e3), or max intensity projection (e4–e6) where the DVE is arrested at the distal tip after 8 h culture with 10 nM thapsigargin. **(B)** Example of DMSO control embryos successfully migrated after 8 h. **(C, D)** Example whole-mount immunohistochemistry staining of cell proliferation marker, Phospho-HistoneH3 (pHH3), and apoptotic marker Cleaved Caspase-3 (Casp3) in cultured embryos. **(C′–D′)** Quantification of cell proliferation and cell apoptosis showed no significant different between controls (*N* = 7) and cultured embryos (*N* = 7) (Student *T* test, *p* ≥ 0.05). **(E)** Embryos cultured in 10 nM thapsigargin (*N* = 34) for 8 h were slightly smaller than DMSO (*N* = 27) control cultured embryos (Student *T* test: Proximal-distal length *p* ≤ 0.01, embryonic width *p* ≤ 0.05, extraembryonic width *p* ≤ 0.05). Tabulated data for C–E can be found in S4 Data.
(TIF)

S5 Fig Whole-mount immunofluorescence of cell fate markers and F-actin in thapsigargin-cultured E5.5 embryos. Hex-GFP embryos cultured for 8 h in the SERCA inhibitor thapsigargin or DMSO control and stained for **(A)** epiblast marker OCT4 (DMSO *N* = 4, 10 nM thapsigargin *N* = 6), **(B)** extraembryonic ectoderm markers CDX2 (DMSO *N* = 5, 10 nM thapsigargin *N* = 4), no difference was seen in either marker. **(C)** Example embryo showing phalloidin staining on cultured embryos showing that the F-actin cytoskeleton is unaffected in thapsigargin cultured embryos (*N* = 18), compared to DMSO (*N* = 15) and that VE cells remain as a monolayer epithelium. Max-intensity projection (MIP).
(TIF)

S1 Table Live imaging experiments to analyze calcium activity in the GCaMP6f mouse line. Table detailing live imaging experiments of the calcium reporter mouse line GCaMP6f from E0.5-E5.5. The table details microscope imaging parameters, number of embryos imaged, the incidence of calcium transients, and reference to supporting movies. Data from these experiments are presented in Figs 1–4, as referenced in the text.
(XLSX)

S1 Data The data underlying Fig 2E–2H. Tables detailing the analysis of cytoplasmic calcium transients in *N* = 14 E5.5 GCaMP6f embryos imaged at a mid-sagittal optical plane every 10 s for 10 min. Calcium transients were analyzed by tissue; epiblast, extraembryonic ectoderm (EXE), visceral endoderm overlying the epiblast (emVE), and visceral endoderm overlying the EXE (exVE).
(XLSX)

S2 Data The data underlying Fig 3E–3G. Table detailing the analysis of cytoplasmic calcium transients in *N* = 14 E5.5 GCaMP6f embryos imaged at a mid-sagittal optical plane every 10 s for 10 min. Cells from all tissues were pooled together and analyzed based on their dynamics, using an unbiased clustering-based method, HDBSCAN (see Methods), identifying three clusters numbered cluster 1–3. Note that in the table, not all transients will have an oscillation period as it requires cells to have a second transient within the 10-min imaging period. These single transient events are therefore marked as N/A in the calcium oscillation period column.
(XLSX)

S3 Data The data underlying Fig 5A and 5B. Table detailing the analysis of cytoplasmic calcium transients in E5.5 GCaMP6f embryos cultured with SERCA inhibitors thapsigargin (10 nM), CPA (200 nM), or DMSO as a solvent control. A mid-sagittal section of each embryo was imaged at a 10-s interval for 2 min, and the total number of oscillations counted at baseline (t0), and 30 min post-treatment. For CPA experiments, embryos were also washed in fresh media and allowed to recover for an additional 30 min (“recovery”).
(XLSX)

S4 Data The data underlying S4C–S4E Fig. Table detailing of E5.5 embryos treated for 8 h with 10 nM thapsigargin. Embryos were analyzed for cell division events by staining for phospho-Histone H3, apoptosis by staining for Cleaved Caspase-3. The proximal-distal length, embryonic and extraembryonic width of each embryo was measured post-culture.
(XLSX)

S1 Movie E0.5 GCaMP6f live imaging. E0.5 GCaMP6f:CD1 mouse embryo imaged using a ZEISS LSM 880 confocal microscope at 20 min intervals for a short timelapse of; 10-s interval for 5 min.
(MOV)

S2 Movie E1.5 GCaMP6f live imaging. E1.5 GCaMP6f:mTmG mouse embryo imaged using a ZEISS LSM 880 confocal microscope at 20-min intervals for a short timelapse of; 10-s interval for 5 min.
(MOV)

S3 Movie E1.5 GCaMP6f z-stack fly-through. E1.5 GCaMP6f:mTmG mouse embryo imaged using a ZEISS LSM 880 confocal microscope showing a fly-through of optical z-sections in a live embryo.
(AVI)

S4 Movie E1.5 GCaMP6f live imaging—10 min duration. E1.5 GCaMP6f:mTmG mouse embryo imaged using a ZEISS LSM 880 confocal microscope every 5 s for 10 min.
(AVI)

S5 Movie E2.5 GCaMP6f live imaging. E2.5 GCaMP6f:mTmG mouse embryo imaged using a ZEISS LSM 880 confocal microscope every 5 s for 10 min.
(AVI)

S6 Movie E3.0 GCaMP6f live imaging—example embryo 1. E3.0 GCaMP6f:mTmG mouse embryo imaged using a ZEISS LSM 880 confocal microscope every 5 s for 10 min.
(AVI)

S7 Movie E3.0 GCaMP6f live imaging—example embryo 2. E3.0 GCaMP6f:mTmG mouse embryo imaged using a ZEISS LSM 880 confocal microscope every 5 s for 10 min.
(AVI)

S8 Movie E4.75 GCaMP6f live imaging. E4.75 GCaMP6f:mTmG mouse embryo imaged using a ZEISS LSM 880 confocal microscope every 5 s for 10 min.
(AVI)

S9 Movie E5.5 GCaMP6f live imaging—example embryo 1. E5.5 GCaMP6f:mTmG mouse embryo imaged using a ZEISS LSM 880 confocal microscope every 5 s for 10 min.
(AVI)

S10 Movie E5.5 GCaMP6f live imaging—example embryos. Three E5.5 GCaMP6f:mTmG mouse embryos imaged using a ZEISS LSM 880 confocal microscope every 5 s for 10 min.
(AVI)

S11 Movie E5.5 GCaMP6f live imaging while attached to maternal decidual tissue. E5.5 GCaMP6f:CD1 mouse embryo still attached to maternal tissue (decidual endometrium) and still surrounded by the extraembryonic Reichert’s membrane showing that widespread calcium oscillations exist prior to complete removal of the embryos from their uterine environment.
(AVI)

S12 Movie E5.5 GCaMP6f live imaging—Z.1 light-sheet. E5.5 GCaMP6f:mTmG mouse embryo imaged using a ZEISS Z.1 light-sheet microscope. A z-stack volume was acquired every 10 s for a duration of 6 min and visualized as a max intensity projection (MIP).
(AVI)

S13 Movie E5.5 GCaMP6f live imaging—lattice light-sheet. E5.5 GCaMP6f:mTmG mouse embryo imaged using a ZEISS lattice light-sheet 7 microscope. A near full z-stack volume was acquired every 20 s for a duration of 20 min and visualized as a max intensity projection (MIP).
(AVI)

S14 Movie E5.5 GCaMP6f live imaging—lattice light-sheet, calcium wave. E5.5 GCaMP6f:mTmG mouse embryo imaged using a ZEISS lattice light-sheet 7 microscope. A near full z-stack volume was acquired every 20 s for a duration of 20 min and visualized as a max intensity projection (MIP). A subset of the movie is shown here, capturing a wave of calcium spreading from an emVE into neighboring epiblast cells.
(AVI)

S15 Movie E5.5 GCaMP6f live imaging—lattice light-sheet, apical to basal calcium wave. E5.5 GCaMP6f:mTmG mouse embryo imaged using a ZEISS lattice light-sheet 7 microscope. A near full z-stack volume was acquired every 2 s for a duration of 20 min and visualized as a max intensity projection (MIP). A sub-set of the movie is shown here, capturing a wave of calcium in epiblast cells that moves from the apical, to the basal aspect of epiblast cells.
(AVI)

S16 Movie E5.5 Hex-GFP live imaging—culture with DMSO or thapsigargin. E5.5 Hex-GFP:CD1 mouse embryos cultured with DMSO, or 10 nM thapsigargin an inhibitor of SERCA and imaged using a ZEISS lattice light-sheet 7 microscope at a 5 min interval for 6 h. Hex-GFP marks the migratory DVE cell population. In DMSO treatment, DVE cells migrate correctly, but when embryos are cultured with thapsigargin they remain arrested at the distal tip of the embryo. Interestingly, though DVE cells do not migrate when cultured with thapsigargin, they still retain highly dynamic cellular projections.
(AVI)

## Abbreviations

BSA: Bovine Serum Albumin
CNN: convolutional neural network
CPA: cyclopiazonic acid
DAPI: 4′,6-diamidino-2-phenylindole
DMSO: dimethyl sulfoxide
DVE: distal visceral endoderm
ER: endoplasmic reticulum
HDBSCAN: hierarchical density-based clustering method
LLSM: Lattice light-sheet microscopy
MARVs: Membrane-associated RNA-containing Vesicles
MIPs: max intensity projections
PBS: phosphate-buffered saline
PBT: Tween-20 in PBS
ROI: regions of interest
SERCA: sarcoplasmic/endoplasmic reticulum Ca^2+^ ATPase
VE: visceral endoderm
