# Supplementary figures and images for: Calcium signaling is required for anterior patterning in the mouse embryo
Source: PLoS Biol. 2025 Oct 6;23(10):e3003430. doi: 10.1371/journal.pbio.3003430 (PMC12548847; doi:10.1371/journal.pbio.3003430)

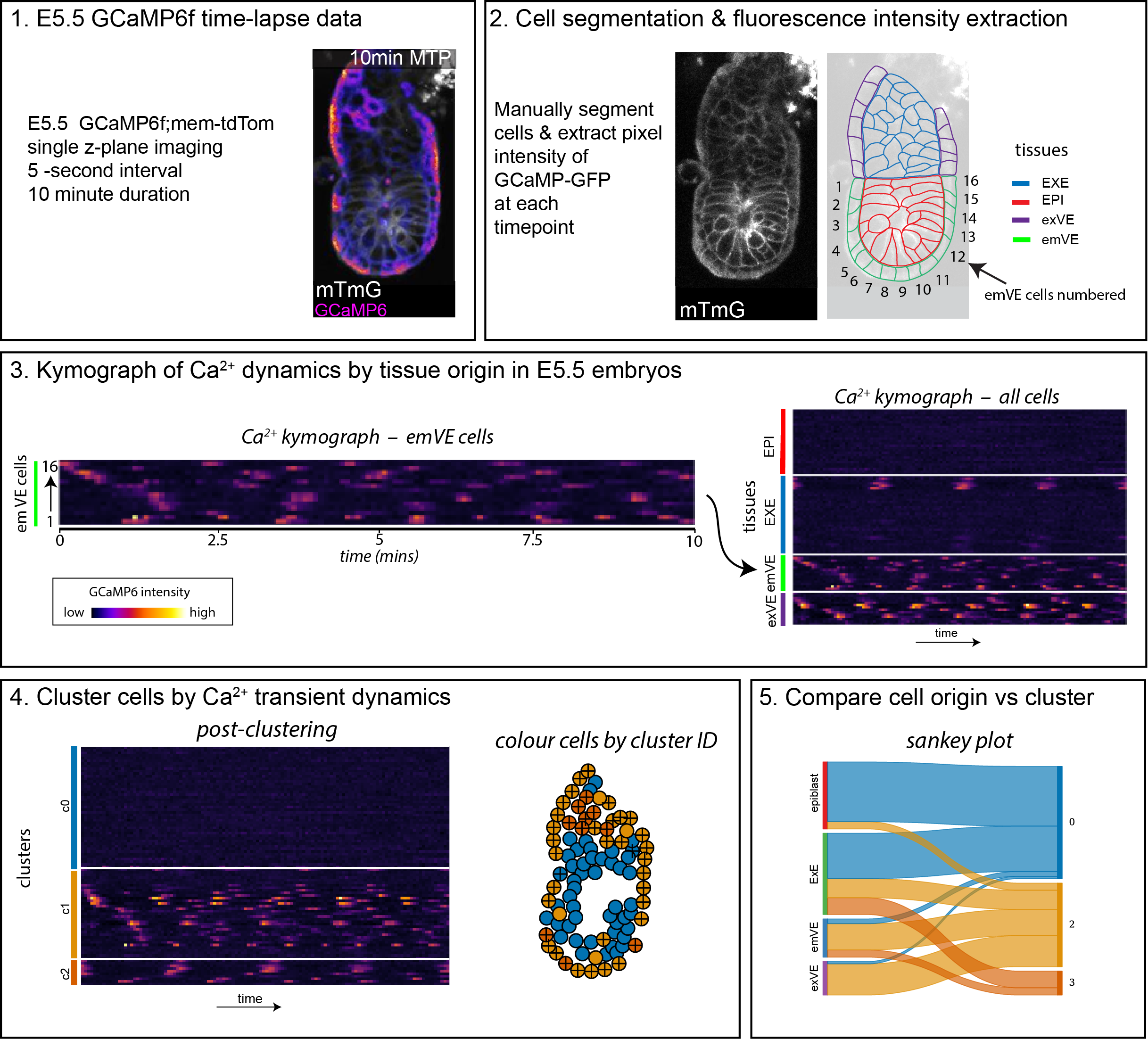

Supplement: S1 Fig — (1) E5.5 GCaMP6f:membrane-tdTomato embryos (N = 14) were imaged at a single z-plane for 5-s interval for 10 min. (2) Each cell was manually segmented and given an ID number. Average pixel intensity for each cell was extracted at every time point. (3) GCaMP6f intensity for each embryo was plotted as a kymograph with cells ordered by tissue along the y-axis and 5-s interval time points along the x-axis. (4). Traces of each cell were pairwise compared to construct a similarity matrix where similarity was defined as the largest positive value of the signal cross-correlation. A hierarchical density-based clustering method, HDBSCAN [46] was then applied to automatically generate clusters. Kymographs were re-plotted to reflect the unbiased clustering and a spatial map of the embryo was colored according to the clusters—showing here clustering results of a single embryo. (5) A Sankey plot was generated for each embryo to show the contribution of cells from tissues to each cluster. (TIF) [file pbio.3003430.s001.tif]

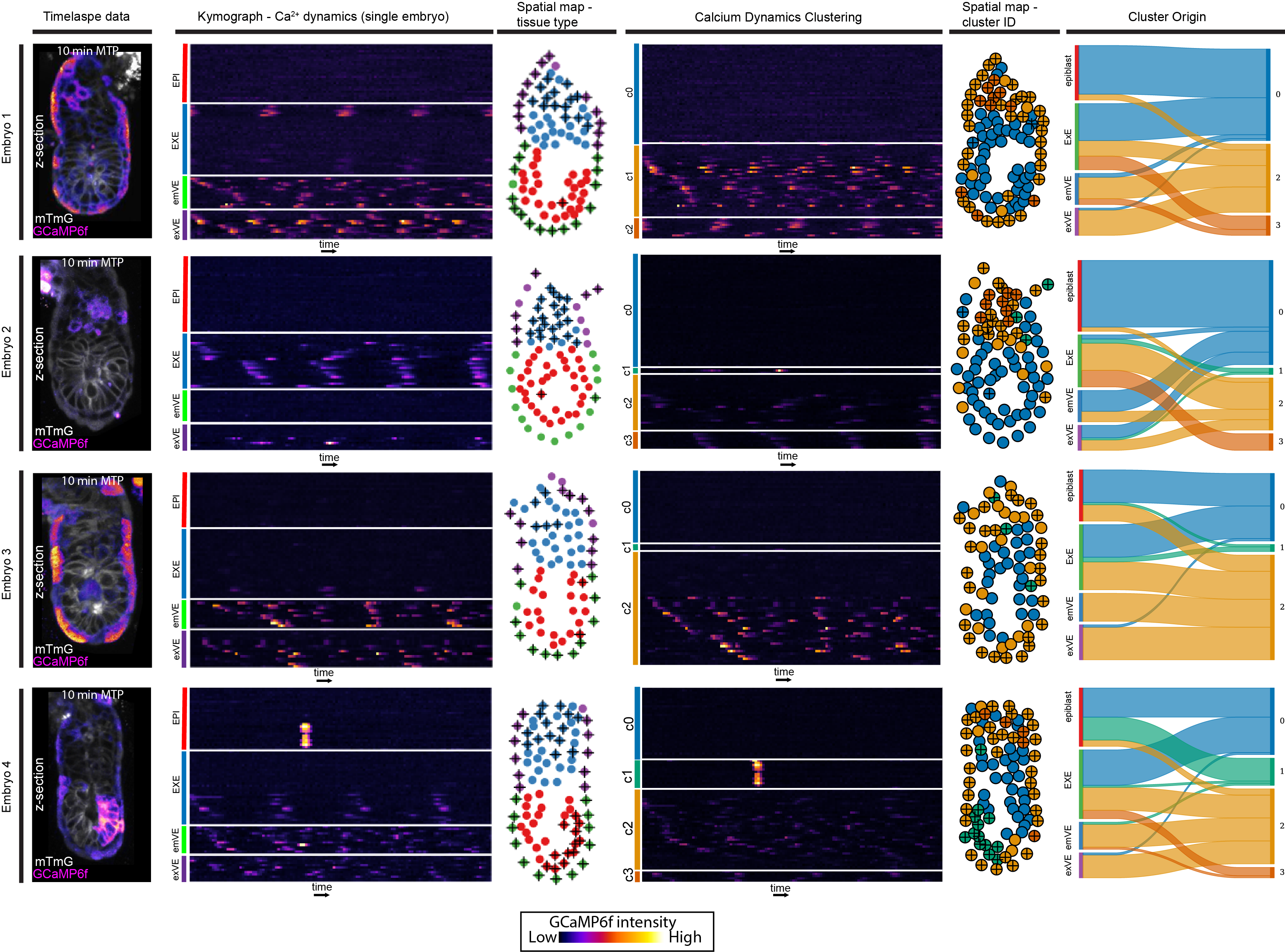

Supplement: S2 Fig — E5.5 GCaMP6f:membrane-tdTomato embryos imaged at a single z-plane for 5-s interval for 10 min. First column shows max-intensity time projection (MTP) of 10 min timelapse. For each embryo, a kymograph and spatial map are shown ordered first by tissue type, then after global clustering. In the final column a Sankey graph shows the contribution of cells in each tissue to the clusters. (TIF) [file pbio.3003430.s002.tif]

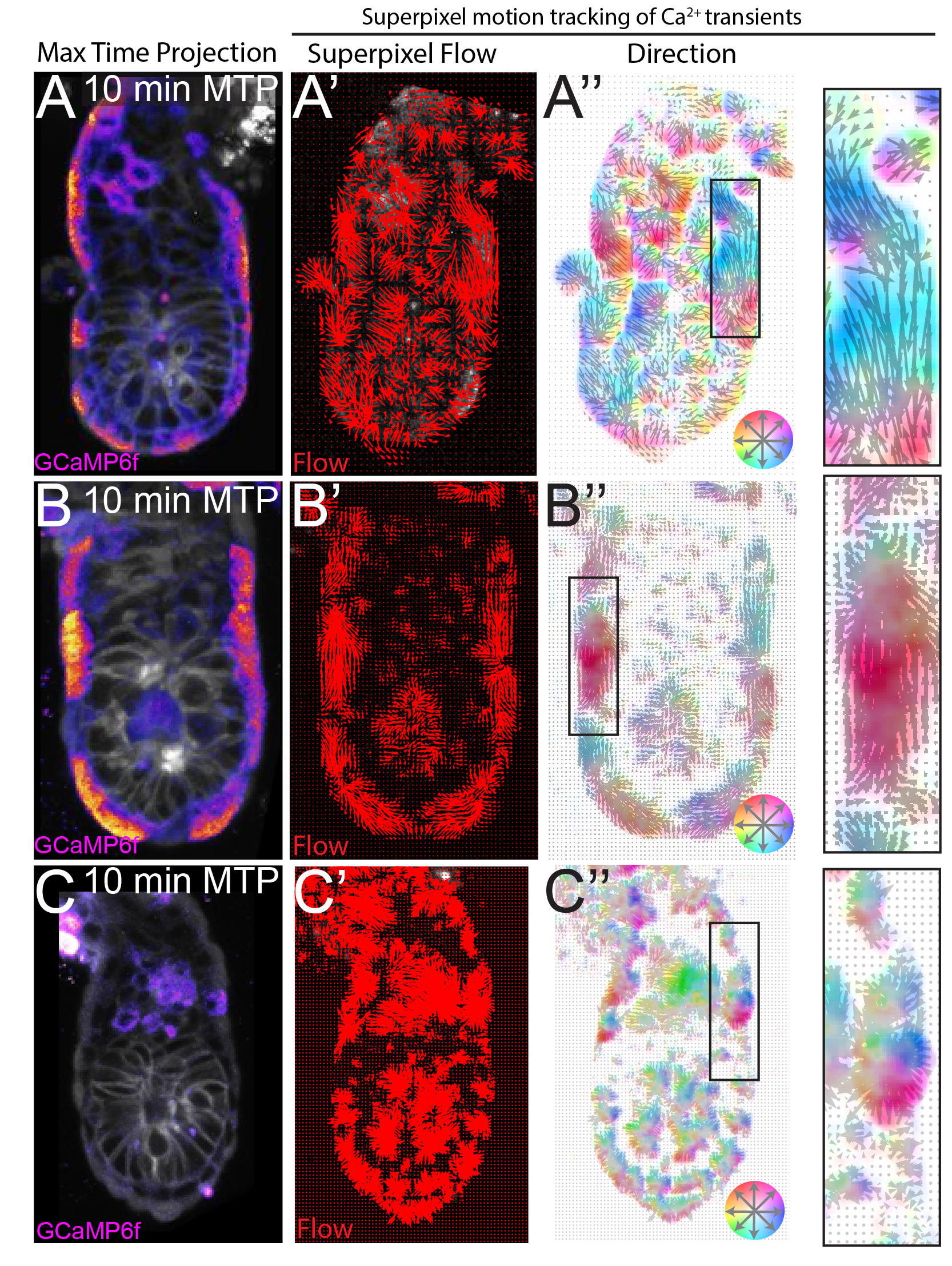

Supplement: S3 Fig — (A–C) Selected examples of E5.5 GCaMP6f:membrane-tdTomato embryos imaged every 5 s for 10 min. (A′, B′, C′) Motion Ca2+ transient flow using super pixel tracking. Red arrows represent the local directional motion of the superpixel during 10 min imaging duration. (A″, B″, C″) Ca2+ transient motion tracking outputs color-coded by vector direction, showing intercellular Ca2+ transients can move proximally or distally along the VE. (TIF) [file pbio.3003430.s003.tif]

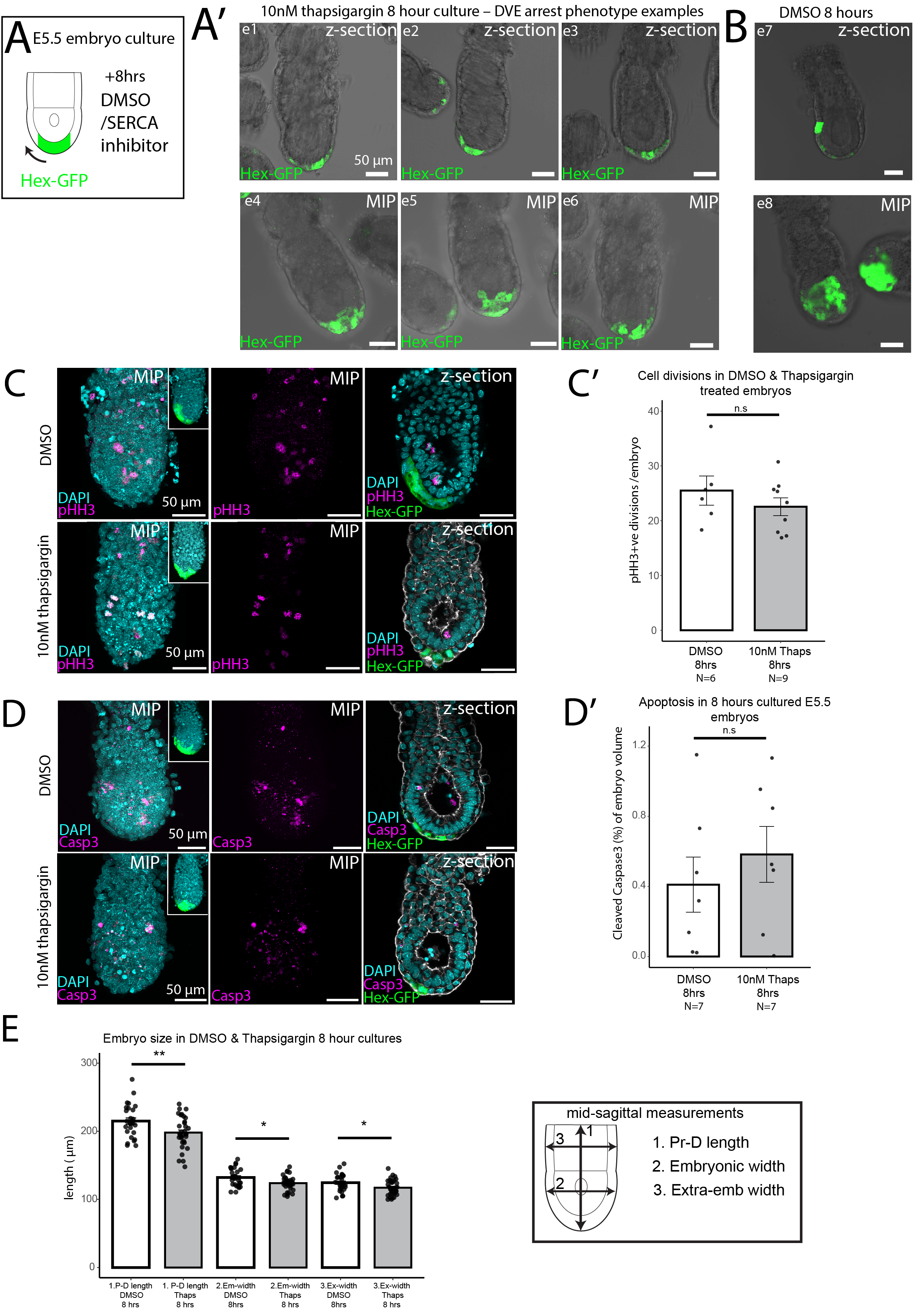

Supplement: S4 Fig — (A) Hex-GFP embryos were cultured for 8 h in the SERCA inhibitor thapsigargin. (A′–A″) Additional examples of Hex-GFP embryos showing a single z-section (e1–e3), or max intensity projection (e4–e6) where the DVE is arrested at the distal tip after 8 h culture with 10 nM thapsigargin. (B) Example of DMSO control embryos successfully migrated after 8 h. (C, D) Example whole-mount immunohistochemistry staining of cell proliferation marker, Phospho-HistoneH3 (pHH3), and apoptotic marker Cleaved Caspase-3 (Casp3) in cultured embryos. (C′–D′) Quantification of cell proliferation and cell apoptosis showed no significant different between controls (N = 7) and cultured embryos (N = 7) (Student T test, p ≥ 0.05). (E) Embryos cultured in 10 nM thapsigargin (N = 34) for 8 h were slightly smaller than DMSO (N = 27) control cultured embryos (Student T test: Proximal-distal length p ≤ 0.01, embryonic width p ≤ 0.05, extraembryonic width p ≤ 0.05). Tabulated data for C–E can be found in S4 Data. (TIF) [file pbio.3003430.s004.tif]

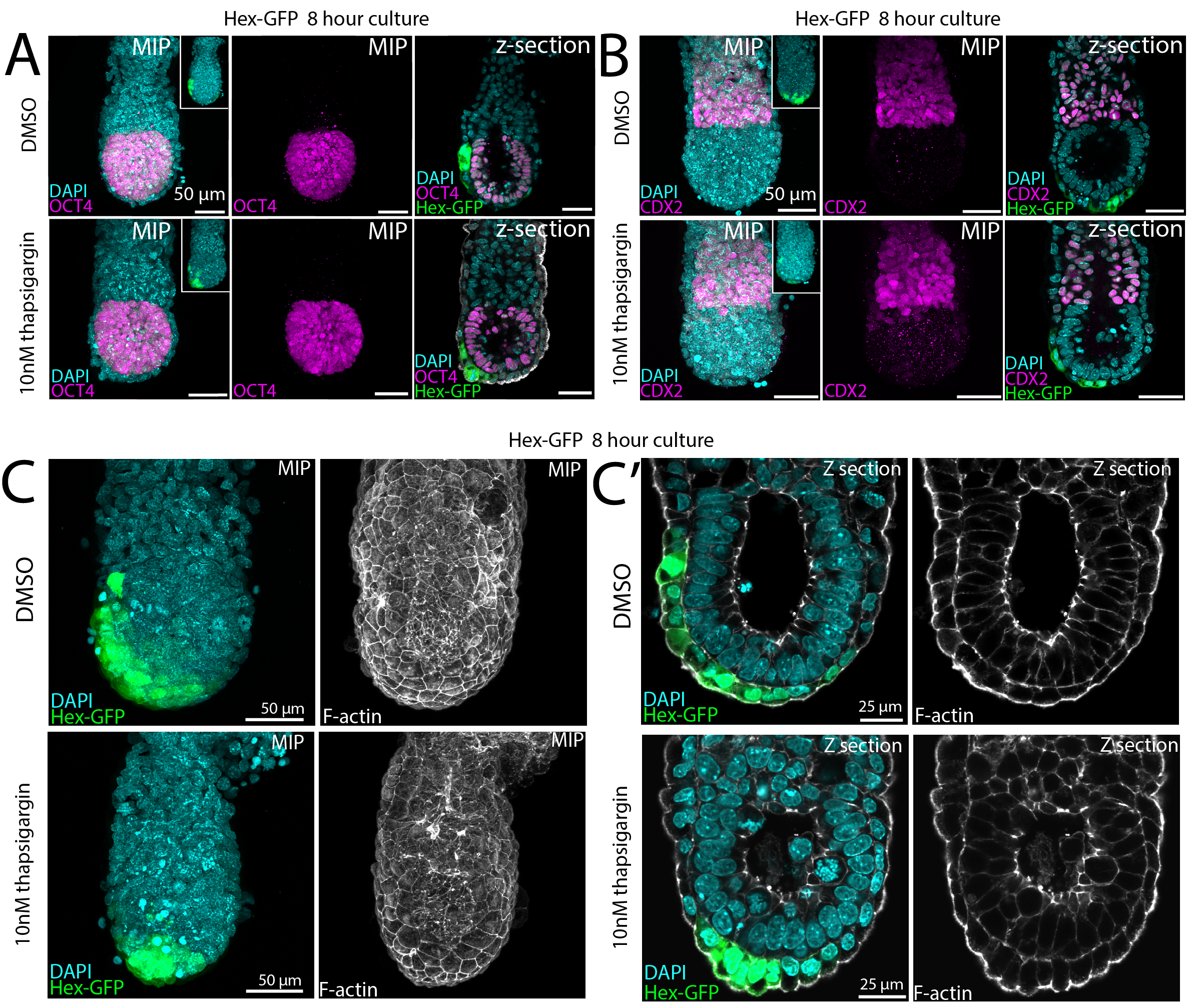

Supplement: S5 Fig — Hex-GFP embryos cultured for 8 h in the SERCA inhibitor thapsigargin or DMSO control and stained for (A) epiblast marker OCT4 (DMSO N = 4, 10 nM thapsigargin N = 6), (B) extraembryonic ectoderm markers CDX2 (DMSO N = 5, 10 nM thapsigargin N = 4), no difference was seen in either marker. (C) Example embryo showing phalloidin staining on cultured embryos showing that the F-actin cytoskeleton is unaffected in thapsigargin cultured embryos (N = 18), compared to DMSO (N = 15) and that VE cells remain as a monolayer epithelium. Max-intensity projection (MIP). (TIF) [file pbio.3003430.s005.tif]
